# Efficacy and safety of PD-1/PD-L1 and CTLA-4 immune checkpoint inhibitors in the treatment of diffuse pleural mesothelioma: a systematic review and meta-analysis

**DOI:** 10.3389/fimmu.2025.1578746

**Published:** 2025-09-02

**Authors:** Peiyuan Sun, Dandan Song, Ning Ma, Shufu Hou, Lei Liu, Jing Gao, Yanyan Tian

**Affiliations:** ^1^ Department of Respiratory Medicine, Shandong Provincial Third Hospital, Shandong University, Jinan, China; ^2^ Department of Neurology, Shandong Provincial Third Hospital, Shandong University, Jinan, China; ^3^ Department of Gastrointestinal Surgery, Jiaozhou Central Hospital, Qingdao, Shandong, China; ^4^ Department of Gastrointestinal Surgery, Central Hospital Affiliated to Shandong First Medical University, Jinan, China; ^5^ Shandong Provincial Third Hospital, Shandong University, Jinan, China; ^6^ Department of Neurosurgery, Shandong Provincial Third Hospital, Shandong University, Jinan, China

**Keywords:** PD-1, PD-L1, CTLA-4, immune checkpoint inhibitors, malignant pleural mesothelioma

## Abstract

**Background:**

While clinical trials confirm the therapeutic value of PD-1/PD-L1 and CTLA-4 inhibitors in diffuse pleural mesothelioma, their real-world safety and efficacy profiles remain incompletely defined. This meta-analysis synthesizes clinical evidence to comprehensively evaluate these outcomes, addressing an urgent need for robust real-world evidence.

**Methods:**

PubMed, Embase, the Cochrane Library were systematically searched for relevant studies. Outcomes including median progression-free survival (mPFS), median overall survival (mOS), 1-year overall survival (1-y OS), 1 year progression-free survival (1-year PFS),objective response rate (ORR), disease control rate (DCR), complete response(CR),partial response (PR), stable disease(SD), progressive disease(PD), treatment-related adverse events (TRAEs) and ≥grade 3 TRAEs were extracted for further analysis. The risk of bias was assessed by subgroup analysis.

**Results:**

14 articles with 1345 patients were identified and subjected to meta-analysis. With regard to survival analysis, the pooled mOS and mPFS were 6.66 months (95%CI 4.85-9.16) and 2.92 months (95%CI 2.23-3.83), respectively. In terms of tumor response, the pooled ORR and DCR were 21% (95%CI 6%-41%) and 49% (95%CI 27%-71%), respectively. The pooled AEs rate and ≥ grade 3 AEs rate were 94% (95%CI 86%-99%) and 44% (95%CI 30%-58%).

**Conclusion:**

PD-1/PD-L1 inhibitors combined with CTLA-4 inhibitors have shown effective clinical responses in the treatment of Diffuse Pleural Mesothelioma (DPM). Although the incidence of adverse reactions is high, they are generally tolerable.

**Systematic Review Registration:**

www.inplasy.com, identifier INPLASY202520045.

## 1 Introduction

Diffuse pleural mesothelioma (DPM), a rare but highly aggressive neoplasm arising from the mesothelial lining of the pleural cavity, is characterized by insidious onset and dismal prognosis, with a documented 5-year survival rate below 10% across major epidemiological studies (1–3). The predominant clinical manifestations in early disease stages include progressive pleuritic chest pain exacerbated by respiratory movements or positional changes, accompanied by dyspnea secondary to pulmonary parenchymal compression and pleural effusion accumulation (4, 5). Asbestos exposure remains the principal etiological determinant, with a characteristic latency period spanning 20–50 years between initial exposure and clinical diagnosis (6, 7). Despite global asbestos restriction policies (8), persistent mining activities in developing economies and prolonged disease latency contribute to geographically heterogeneous incidence patterns, with rising trends observed in multiple regions (9).

Histologically, DPM demonstrates three distinct subtypes: epithelioid (60-70% of cases, associated with relatively favorable prognosis), biphasic (20-35%), and sarcomatoid (10-15%, correlating with aggressive clinical course) (10–12). Significant gender disparities exist, with male predominance in incidence (male:female ratio ≈3:1) contrasting with superior survival outcomes in female patients (13). Current management employs multimodal strategies integrating cytoreductive surgery, platinum-based chemotherapy, radiotherapy, and emerging immunotherapies (14, 15). Recent data from the MARS2 trial challenge the role of cytoreductive surgery, demonstrating no survival benefit for pleurectomy/decortication compared to systemic therapy alone. Consequently, multimodal strategies now prioritize systemic therapies including immunotherapy. Nevertheless, therapeutic efficacy remains suboptimal, as evidenced by population-based registry data demonstrating median overall survival (OS) of 9.9-10.3 months across international cohorts (16, 17). The clinical challenge is compounded by frequent late-stage diagnosis precluding surgical intervention and high recurrence rates post-resection, even in early-stage operable cases (18). In 2004, the cisplatin-pemetrexed regimen became a first-line standard for unresectable disease based on EMPHACIS trial evidence (14). Subsequent phase III trials (MAPS study) demonstrated a modest 2.7-month OS improvement with bevacizumab augmentation of this backbone regimen (19). The persistent therapeutic limitations underscore the imperative for novel treatment modalities. The advent of immune checkpoint inhibitors has revolutionized DPM management, particularly targeting the PD-1/PD-L1 and CTLA-4 pathways. Mechanistically, PD-1/PD-L1 interaction facilitates tumor immune evasion through T-cell anergy induction, while CTLA-4 modulates early T-cell activation via competitive CD80/86 binding (20, 21). The non-redundant nature of these pathways provides a rationale for combinatorial blockade to achieve synergistic anti-tumor immunity (22), an approach validated in multiple malignancies (23–25). The landmark CheckMate 743 phase III trial demonstrated superior OS with nivolumab/ipilimumab dual checkpoint inhibition versus chemotherapy (median OS 18.1 vs. 14.1 months; HR 0.74, 95%CI 0.60-0.91; p=0.002), leading to FDA/EMA approval for first-line unresectable DPM (26–29). This paradigm shift established immunotherapy as a new standard of care.

Recent observational studies challenge the real-world generalizability of clinical trial outcomes. Schmid et al. reported suboptimal efficacy and unmitigated toxicity profiles with dual checkpoint inhibition compared to conventional regimens (30). This efficacy discordance between controlled trials and real-world practice may stem from differences in patient selection criteria, comorbidity burden, and treatment protocols. To resolve these controversies and comprehensively evaluate therapeutic efficacy, we conducted a systematic meta-analysis synthesizing all available clinical evidence on dual immune checkpoint inhibition in DPM. This investigation aims to clarify the risk-benefit profile of this therapeutic strategy across diverse clinical contexts.

## 2 Methods

### 2.1 Article searching

This meta-analysis strictly followed the guidelines specified in the Preferred Reporting Items for Systematic Reviews and Meta Analyses (PRISMA) checklist (31).The PRISMA checklist ensures a comprehensive and transparent reporting of systematic reviews and meta-analyses, emphasizing methodological clarity and quality. The study protocol has been registered with the International Platform of Registered Systematic Review and Meta-analysis Protocols (INPLASY) (Registration ID: INPLASY202520045). Online databases (Cochrane Library, Embase, and PubMed) were searched for relevant clinical trials published from the establishment of these databases through November 30, 2024. Search terms included: “anti-PD-1”, “anti-PD-L1”, “anti-CTLA-4”, “immune checkpoint inhibitors”,”nivolumab”, “ipilimumab”, “tremelimumab”,”durvalumab”, AND “pleural mesothelioma”, “malignant pleural mesothelioma”, “ diffuse pleural mesothelioma”, “malignant pleural mesothelioma”,”MPM” and”DPM”. In addition to utilizing free search terms and Medical Subject Headings (MeSH) for searching within titles or abstracts, we screened the references of selected articles to ensure comprehensive retrieval. In cases of duplicate publications, more comprehensive studies were chosen for subsequent meta-analysis. All information was extracted by 2 authors independently, and any consensus was resolved through negotiation.

### 2.2 Study selection

Obtained records were exported to EndNote software (Clarivate Analytics, Philadelphia, PA, USA).After removing the duplicate publications, two review authors independently reviewed the title/abstract of the articles according to the inclusion and exclusion criteria. Afterward, the same two authors screened the full-texts of the selected records, independently. Discrepancies were resolved by consulting a third author.

### 2.3 Eligibility criteria

Trials were included if the following criteria were met (1): adults (≥18 years)had a histologically proven diagnosis of diffuse pleural mesothelioma with locally advanced or metastatic disease; (2):a PD-1/PD-L1 and CTLA-4 inhibitors with or without other standard treatments was given to one of the study arms; and (3):outcomes of interest in terms of efficacy (i.e. median overall survival (mOS), median progression-free survival (mPFS), 1-year overall survival (1-y OS), 1 year progression-free survival (1-year PFS),objective response rate (ORR), disease control rate (DCR), complete response(CR),partial response (PR), stable disease(SD), progressive disease(PD), and safety (i.e. Treatment - Related Adverse Event(TRAEs) and ≥ grade 3 TRAEs)were reported.

The exclusion criteria were as follows: (1) Patients who do not have diffuse pleural mesothelioma;(2)animal experiments, cell research, reviews, meta-analyses, duplicates, case reports, or letters were not taken into consideration; and (3) studies with patient number less than 10 were excluded. Two investigators independently identified potential eligible articles through inclusion and exclusion criteria. Any disagreement regarding study inclusion was resolved between these two or with a third investigator.

### 2.4 Data extraction and quality assessment

Two researchers conducted independent literature searches, following predetermined criteria and specified strategies. This approach ensures a thorough and unbiased exploration of available literature, utilizing a systematic and structured methodology. Meticulous data extraction was performed, encompassing essential details such as authors, publication year, country, age, sample size, gender, age, stage, median follow-up, drugs, dose, number of treatment lines, research scale, and research methods, including mOS, PFS,1-y OS, 1-y PFS, DCR, ORR, CR,PR,SD,PD,TRAEs、≥ grade 3TRAEs. All included studies were treated as non-randomized trials. The quality of each study was meticulously evaluated using the methodological index for non-randomized studies (MINORS) (32). Studies scoring above 12 points were considered high-quality indicators. This stringent evaluation ensures that only studies meeting robust methodological standards contribute to the overall analysis.

### 2.5 Data synthesis

The primary efficacy endpoint was to estimate the mOS and mPFS after receiving PD-1/PD-L1 inhibitors combined with CTLA-4 inhibitors treatment regimens and the secondary efficacy endpoint was to estimate the pooled rate of 1-y OS, 1-y PFS, DCR, ORR, CR,PR,SD,PD. The safety outcomes were the pooled rates of TRAEs and ≥ grade 3 TRAEs, We used Cochrane’s Q statistic to assess between-study heterogeneity and calculated the Isquare statistic. A random-effect model was applied if obvious heterogeneity was present (I2 >50%), otherwise, a fixed-effect model was chosen (33). The subgroup analysis was conducted according to region(Europe, Other), sample (≥60, <60), Research scale(Single-center, multicenter), Research methods (prospective, retrospective) and Number of treatment lines (1Line, 2Line/latter Line, Any). Differences between groups were tested by the chi-square test. We used STATA version 18.0 (34) to calculate the pooled rates with metaprop command, which requires a nominator and a denominator (which is the total sample size) and some other options like random or fixed effects model. This command was built on the existing Stata command metan, which is routinely used to pool ratios and differences of means (35). A p-value less than 0.05 were treated as statistically significant.

## 3 Results

### 3.1 Study selection and characteristics of the included studies

**Figure 1:**
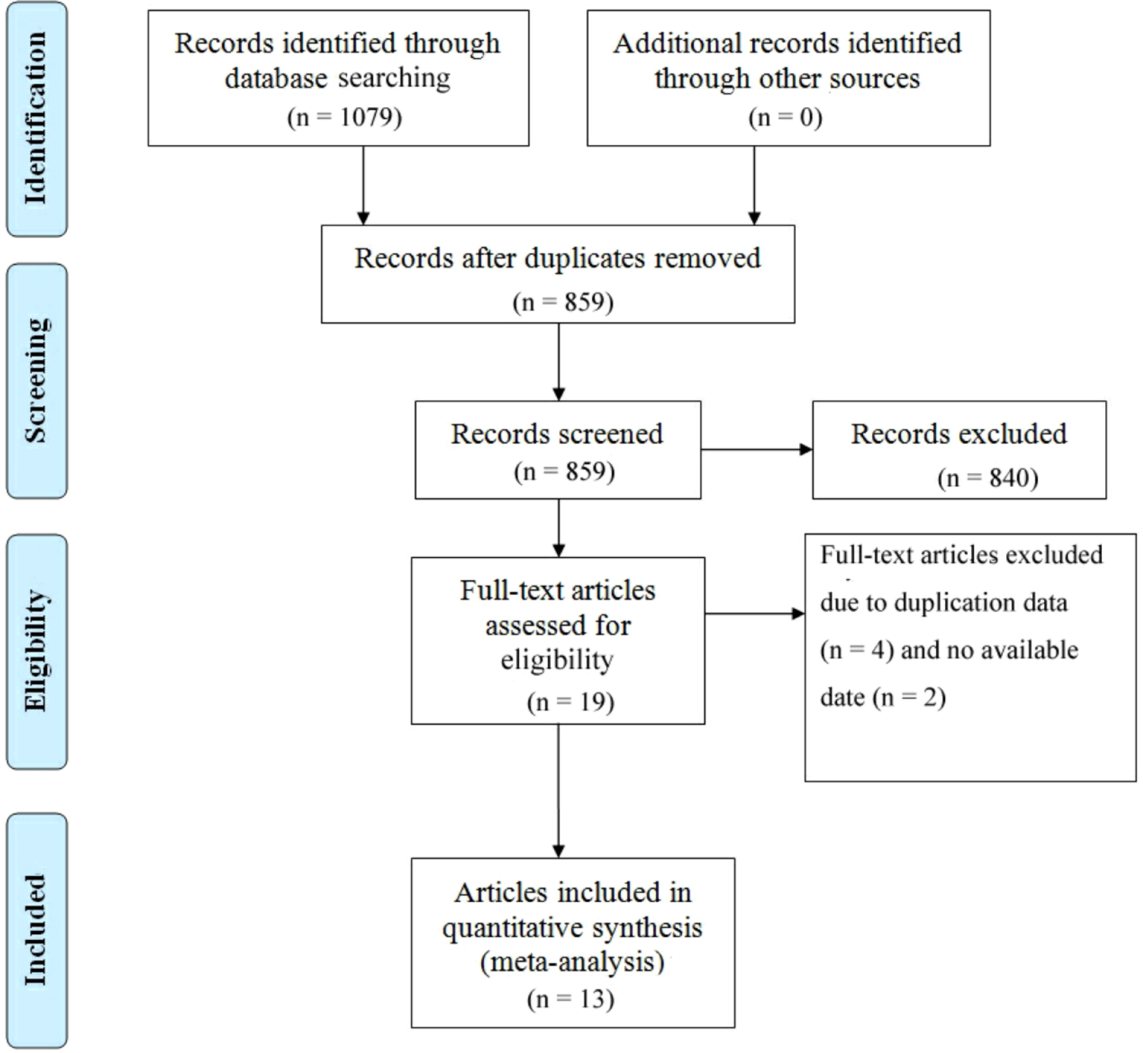
Prisma flowchart illustrating the literature selection process.

There were 1079 documents searched from the databases. Of these, 220 replicated studies were deleted. After reading the title and abstract of each article, 19 articles were screened out. Of those, 4 articles were excluded because of duplicate data, and 2 articles lacking available data were also excluded. Finally, a total of 13 articles meeting the criteria were selected with a total of 1,345 patients (26, 30, 36–46). An article by Schmid includes two cohorts of PD-1 inhibitors plus CTLA-4 inhibitors, one for first-line therapy and the other for second-line therapy (30). We considered the number of treatment lines to be an important source of heterogeneity and therefore discussed them separately. **Figure 1** summarizes the detailed information about article selection. The 6 and 8 included research cohorts were eligible for mOS and mPFS, respectively. The 4、9 and 13 included cohorts were eligible for 1-y OS 、1-y PFS and ORR, respectively. The 12 included cohorts were eligible for CR、PR、DCR、TRAEs and ≥ grade 3 TRAEs data analysis, and 11 were eligible for PD、SD data analysis. **Table 1** lists the characteristics of the 14 cohorts. The extracted characteristics were summarized as follows: authors, publication year, country, age, sample size, gender, age, stage, median follow-up, drugs, dose, number of treatment lines, research scale, and research methods.

**Table 1:** Baseline characteristics of included studies.

Study, year	Country	Age (median)	Sample size	Gender (M/F)	Stage	Median follow-up (months)	Drugs	Dose	Research scale	Research methods	Number of treatment lines	MINORS score
Disselhorst 2019 (36)	the Netherlands	65 (37-79)	35	27/8	I-IV	14.3	Nivolumab+ Ipilimumab	Nivolumab 240 mg every 2 weeks and Ipilimumab 1 mg/kg every 6 weeks	single-center	prospective	2L/later Line	15
Scherpereel 2019 (37)	France	71.2	62	53/9	I-IV	20.1	Nivolumab+ Ipilimumab	3 mg/kg nivolumab every 2 weeks followed by 1 mg/kg ipilimumab every 6 weeks	multicenter	prospective	2L/later Line	15
Baas 2021 (26)	Multicenter	69	303	234/69	I-IV	29.7	Nivolumab+ Ipilimumab	3 mg/kg nivolumab every 2 weeks followed by 1 mg/kg ipilimumab every 6 weeks	multicenter	prospective	1Line	15
Calabro 2021	Italy	64 (53–71)	40	29/11	III-IV	19.2	Durvalumab+ tremelimumab	1 mg/kg tremelimumab and 20 mg/kg durvalumab every 4 weeks	single-center	prospective	Any	14
Lee 2023 (39)	USA	mean:63.4 ± 9.5	11	9/2	I-II	NR	Durvalumab+ tremelimumab	durvalumab (1500 mg) plus tremelimumab (75 mg)	single-center	prospective	1Line	14
Nakamura 2023 (40)	Japan	67 (52–83)	41	34/7	IA-IIIB	10.4	Nivolumab+ Ipilimumab	360 mg nivolumab every 3 weeks and 1 mg/kg of ipilimumab every 6 weeks	single-center	prospective	2L/later Line	14
Bylicki 2024 (41)	France	75	201	160/41	I-IV	18.4	Nivolumab+ Ipilimumab	360 mg nivolumab every 3 weeks and 1 mg/kg of ipilimumab every 6 weeks	multicenter	retrospective	2L/later Line	15
Dumoulin 2024 (42)	the Netherlands	71 (66–76)	184	159/25	NR	12.1	Nivolumab+ Ipilimumab	360 mg nivolumab every 3 weeks and 1 mg/kg of ipilimumab every 6 weeks	multicenter	prospective	Any	15
Enrico 2024 (43)	Multicenter	63 (41-84)	96	57/39	I-IV	24.1	Nivolumab+ Ipilimumab	NR	multicenter	retrospective	1Line	14
Haakensen 2024 (44)	Multicenter	71 (39-83)	118	92/26	NR	17.3	Nivolumab+ Ipilimumab	Nivolumab 240 mg every 2 weeks and Ipilimumab 1 mg/kg every 6 weeks	multicenter	prospective	2L/later Line	14
Kitajima 2024 (45)	Japan	73.5 (63-85)	26	23/3	NR	10	Nivolumab+ Ipilimumab	NR	single-center	retrospective	1Line	14
McNamee 2024 (46)	Australia	72 (19-86)	119	99/20	NR	14.4	Nivolumab+ Ipilimumab	NR	multicenter	retrospective	Any	14
Schmid 2024 1 (30)	Switzerland	77 (62-86)	47	44/3	I-IV	16.6	Nivolumab+ Ipilimumab	NR	multicenter	retrospective	1Line	14
Schmid 2024 2 (30)	Switzerland	68 (49-87)	62	55/7	I-IV	16.6	Nivolumab+ Ipilimumab	NR	multicenter	retrospective	2L/later Line	14

M, male; F, female; NR, not report; Nivolumab; Ipilimumab; Durvalumab; Tremelimumab; Any, including 1 line and 2L/latter Line; MINORS, methodological index for non-randomized studies

### 3.2 Quality assessment

13 non-randomized studies were assessed using the methodological index for non-randomized studies (MINORS), which categorized studies into three dimensions based on eight items, including stated aim, population election, endpoints, and prospective calculation. The quality assessment details are shown in **Table 2**.

**Table 2:** Methodological index for non-randomized studies (MINORS) for quality.

Studies	A clearly stated aim	Inclusion of consecutive patients	Prospective collection of data	Endpoints appropriate to the aim of the study	Unbiased assessment of the study endpoint	Follow-up period appropriate to the aim of the study	Loss to follow up less than 5%	Prospective calculation of the study size	Scores
Disselhorst 2019 (36)	2	2	2	2	2	2	2	1	15
Scherpereel 2019 (37)	2	2	2	2	2	2	2	1	15
Baas 2021 (26)	2	2	2	2	2	2	2	1	15
Calabro 2021	2	2	1	2	2	2	2	1	14
Lee 2023 (39)	2	2	2	2	2	1	2	1	14
Nakamura 2023 (40)	2	2	1	2	2	2	2	1	15
Bylicki 2024 (41)	2	2	1	2	2	2	2	1	15
Dumoulin 2024 (42)	2	2	2	2	2	2	2	1	14
Enrico 2024 (43)	2	2	1	2	2	2	2	1	14
Haakensen 2024 (44)	2	2	1	2	2	2	2	1	14
Kitajima 2024 (45)	2	2	1	2	2	2	2	1	14
McNamee 2024 (46)	2	2	1	2	2	2	2	1	14
Schmid 2024 1 (30)	2	2	1	2	2	2	2	1	14
Schmid 2024 2 (30)	2	2	1	2	2	2	2	1	14

### 3.3 Efficacy

#### 3.3.1 Survival

**Figure 2:**
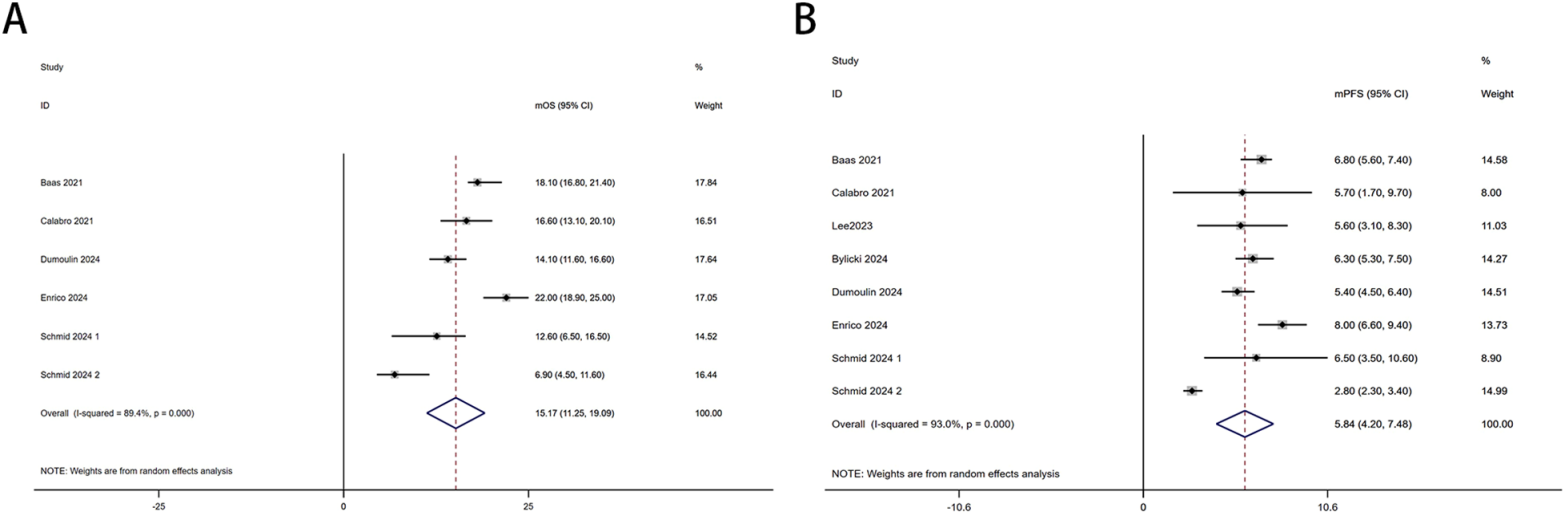
Forest plot for the **(A)**median overall survival (mOS) and **(B)** median progression-free survival (mPFS) in DPM patients receiving PD-1/PD-L1 inhibitors combined with CTLA-4 inhibitors.

**Figure 3:**
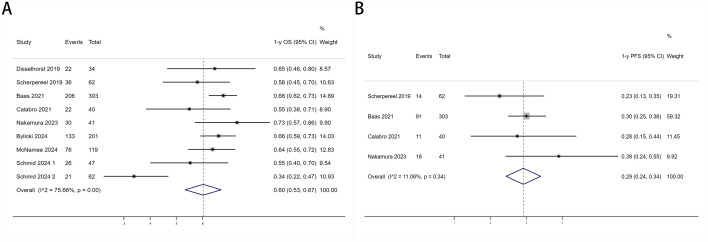
Forest plot for the **(A)**1 year overall survival (1-y OS) and **(B)** 1 year progression-free survival (1-y PFS) in DPM patients receiving PD-1/PD-L1 inhibitors combined with CTLA-4 inhibitors.

Six trials with a total of 732 patients were included to determine the mOS of patients treated with PD-1/PD-L1 inhibitor and CTLA-4 inhibitor. As shown in **Figure 2A**, the random-effect model meta-analysis illuminated that the pooled mOS was 15.17months (95%CI 11.25-19.09,I2 = 89.4%, P<0.0001), suggesting that PD-1/PD-L1 and CTLA-4 immune checkpoint inhibitors achieved good mOS in the treatment of DPM. We also analyzed the mPFS of PD-1/PD-L1 inhibitor and CTLA-4 inhibitor in DPM. As shown in **Figure 2B**, the pooled mPFS of 944 patients in 8 studies was 5.84months (95%CI: 4.20-7.48, I2 = 98.3%, P<0.0001). The result suggests that PD-1/PD-L1 inhibitors and CTLA-4 inhibitors performed well in terms of mPFS in the treatment of DPM. According to **Figure 3A**, the meta - analysis based on the random - effect model demonstrated that the combined 1-y OS rate of nine trials (with 909 patients in total) was 60% (95% CI:53%-67%, I^2^ = 75.66%, P<0.0001). As shown in **Figure 3B**, by using data from four trials involving 446 patients with DPM, the meta - analysis with the fixed - effect model showed that the combined 1 - year PFS rate was 29% (95%CI 24% - 34%,I^2^ = 11.04%,P=0.34).The findings indicate that PD - 1/PD - L1 inhibitors and CTLA - 4 inhibitors had a good performance regarding 1 – y OS and 1 – y PFS when treating DPM.

We performed subgroup analyses based on regions (Europe, other regions), sample sizes (≥60, <60), research scales (single - center, multi - center), research methods (prospective, retrospective), and the number of treatment lines (1Line, 2Line/Later Line, Any) to further analyze PD-1/PD-L1 and CTLA-4 immune checkpoint inhibitors’ efficacy. From **Table 3**, in the subgroup analysis, the DPM patient groups treated with PD - 1/PD - L1 inhibitors and CTLA - 4 inhibitors, including those from Europe, in single - center studies, with a sample size of 60 or more, those in prospective studies, and receiving 1 Line treatment, may benefit more in terms of mOS. As shown in **Table 4**, subgroup analysis revealed that the mPFS of DPM patients receiving PD - 1/PD - L1 inhibitors and CTLA - 4 inhibitors was significantly better in the following scenarios: in other regions, when the sample size was less than 60, in single - center studies, in prospective studies and in 1 Line treatment. Judging from **Table 5**, in the subgroup analysis of the patient groups treated with PD - 1/PD - L1 inhibitors and CTLA - 4 inhibitors, it is indicated that the 1 – y OS of DPM patient groups in the subgroups of other regions, sample size less than 60, single - center studies, prospective studies, and 1Line treatment is higher than that of other subgroups. Because 1-y PFS included few articles, no subgroup analysis was performed.

**Table 3:** Subgroup analysis of pooled of the median overall survival.

Subgroup	No. of studies	Ratio (95% CI)	Heterogeneity	
			I^2^ (%)	Ph
Region				
Europe	4	12.60 (8.53-16.66)	81.4	0.001
Other	2	19.92 (16.10-23.73)	75.0	0.045
Sample				
≥60	4	15.35 (9.92-20.78)	93.4	<0.001
<60	2	15.01 (11.18-18.85)	39.4	0.199
Research scale				
single-center	1	16.60 (13.10-20.10)	–	–
multicenter	5	14.86 (10.16-19.56)	91.5	<0.001
Research methods				
prospective	3	16.27 (13.70-18.84)	62.6	0.069
retrospective	3	13.88 (4.00-23.76)	95.1	<0.001
Number of treatment lines				
1Line	3	17.98 (13.64-22.31)	80.9	0.005
2Line/latter Line	1	6.90 (3.35-10.45)	–	–
Any	2	15.04 (12.67-17.41)	22.9	0.255

**Table 4:** Subgroup analysis of pooled of the median progression-free survival.

Subgroup	No. of studies	Ratio (95% CI)	Heterogeneity	
			I^2^ (%)	Ph
Region				
Europe	6	25% (21%-29%)	9.61	0.35
Other	6	29% (18%-42%)	88.52	<0.001
Sample				
≥60	4	5.57 (3.27-7.88)	95.9	<0.001
<60	4	6.62 (5.81-7.43)	0	0.813
Research scale				
single-center	3	6.63 (5.80-7.46)	0	0.623
multicenter	5	5.70 (3.58-7.82)	94.6	<0.001
Research methods				
prospective	4	6.04 (5.14-6.95)	34.6	0.204
retrospective	4	5.82 (2.95-8.69)	95.6	<0.001
Number of treatment lines				
1Line	4	7.02 (6.21-7.82)	10.2	0.342
2Line/latter Line	2	4.52 (1.09-7.95)	96.8	<0.001
Any	2	5.42 (4.49-6.34)	0	0.886

**Table 5:** Subgroup analysis of pooled of the 1 year overall survival.

Subgroup	No. of studies	Ratio (95% CI)	Heterogeneity	
			I^2^ (%)	Ph
Region				
Europe	6	56% (45%-66%)	76.32	<0.001
Other	3	68% (63%-72%)	–	–
Sample				
≥60	5	59% (49%-69%)	84.77	<0.001
<60	4	62% (53%-71%)	24.27	0.27
Research scale				
Single-center	3	65% (54%-75%)	–	–
multicenter	6	59% (50%-67%)	81.78	<0.001
Research methods				
prospective	4	64% (57%-70%)	29.03	0.24
retrospective	5	59% (47-70%)	83.61	0.01
Number of treatment lines				
1Line	2	66% (61%-71%)	–	–
2Line/latter Line	5	59% (46%-72%)	83.14	<0.001
Any	2	62% (54%-69%)	–	–

#### 3.3.2 Response rates

**Figure 4:**
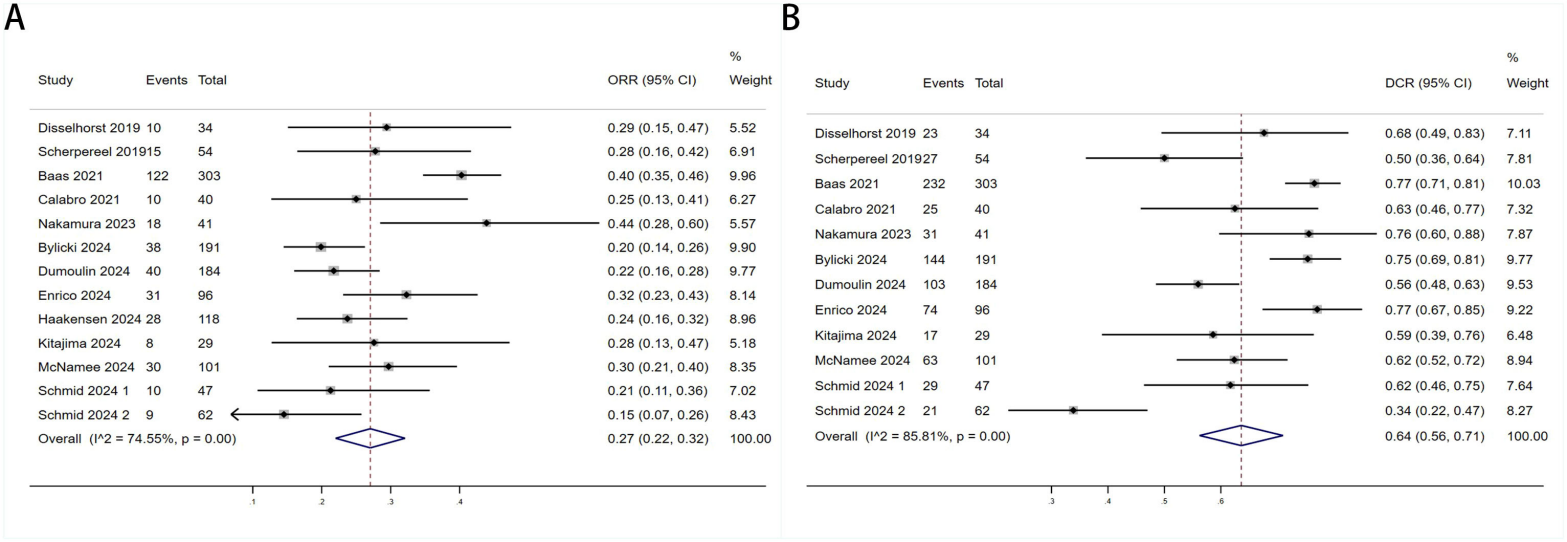
Forest plot for the **(A)** Objective response rate (ORR) and **(B)** Disease control rate (DCR) in DPM patients receiving PD-1/PD-L1 inhibitors combined with CTLA-4 inhibitors.

**Figure 5:**
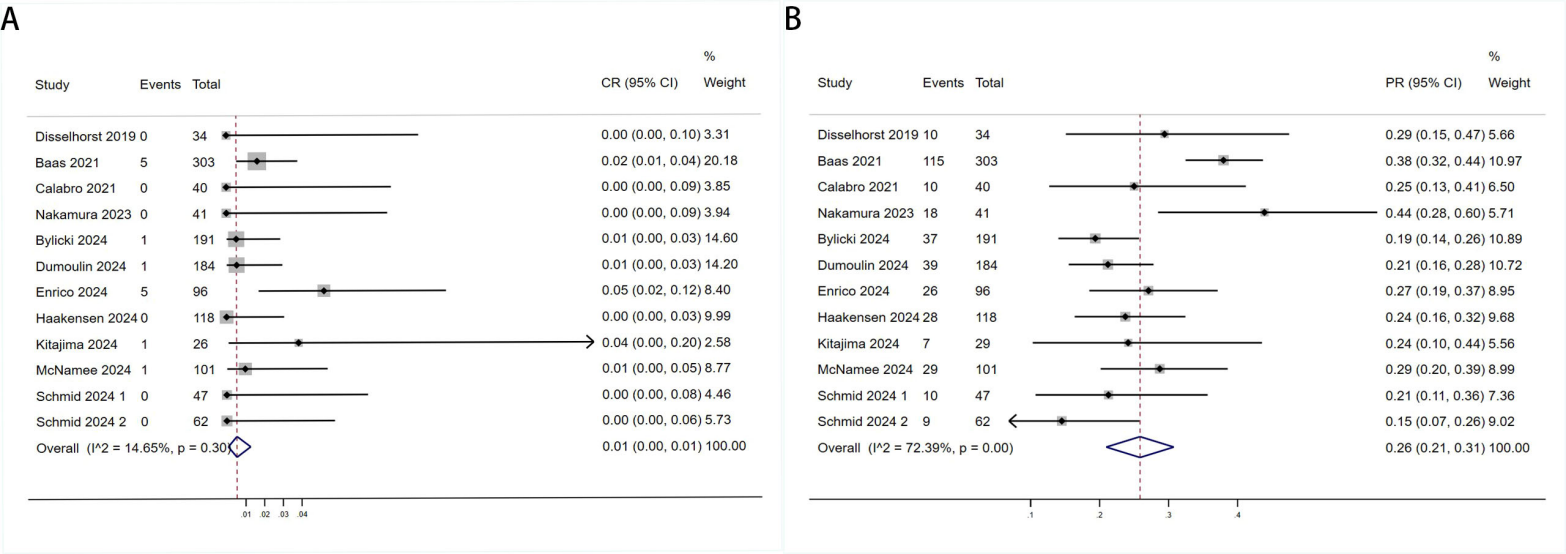
Forest plot for the **(A)** Complete Response (CR) and **(B)** Partial Response (PR) in DPM patients receiving PD-1/PD-L1 inhibitors combined with CTLA-4 inhibitors.

**Figure 6:**
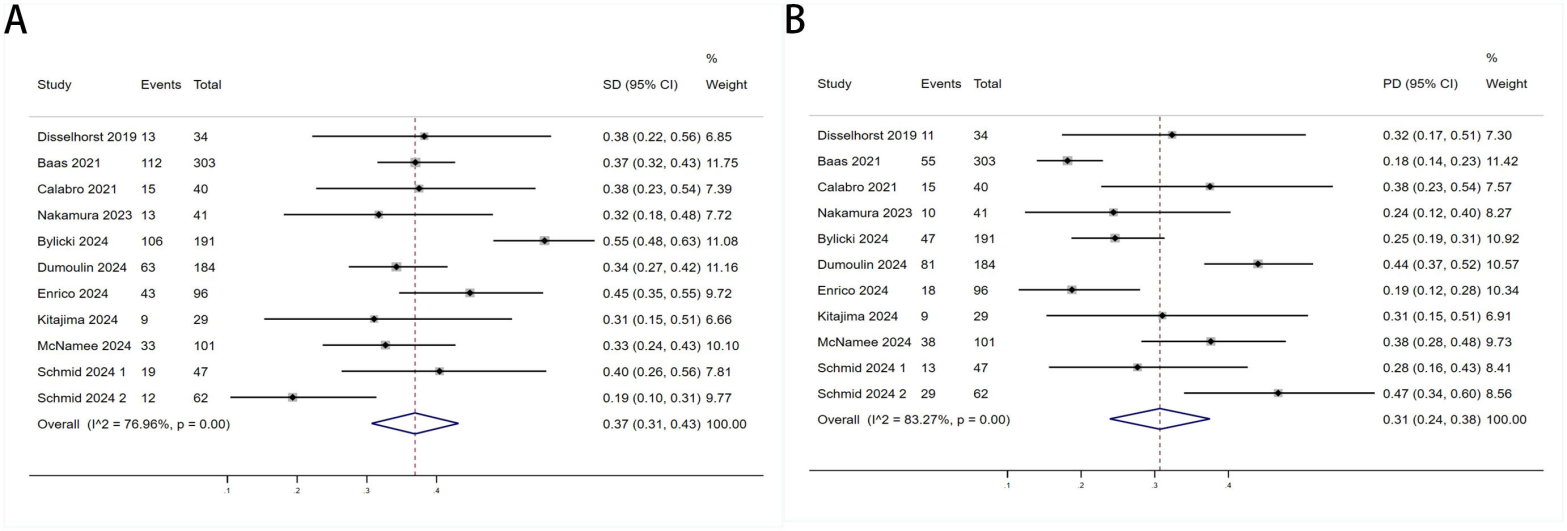
Forest plot for the **(A)** Sta and **(B)** Progressive Disease (PD) in DPM patients receiving PD-1/PD-L1 inhibitors combined with CTLA-4 inhibitors.

Twelve trials with a total of 1300 patients were included to determine the ORR of patients treated with PD-1/PD-L1 inhibitor and CTLA-4 inhibitor. As shown in **Figure 4A**, the random-effect model meta-analysis illuminated that the pooled ORR was 27% (95%CI:22%-32%, I^2^ = 74.55%, P<0.001), suggesting that PD-1/PD-L1 and CTLA-4 immune checkpoint inhibitors achieved good ORR in the treatment of DPM. In terms of DCR, we included 12 studies with 1182 patients. The pooled estimate of DRR was 64%, (95% CI: 56%-71%, I^2^ = 85.81%, P<0.001, **Figure 4B**).It can be indicated from these findings that PD - 1/PD - L1 inhibitors and CTLA - 4 inhibitors performed well in improving the DCR when treating patients with DPM. According to **Figure 5A**, the meta - analysis based on the random - effect model demonstrated that the combined CR of twelve trials (with 1246 patients in total) was 1% (95% CI: 0-1%, I^2^ = 14.65%, P=0.30, **Figure 5A**). Similarly, by using data from twelve trials involving 1246 patients with DPM, the meta-analysis with the random - effect model showed that the combined PR was 26% (95CI: 21% - 31%,I^2^ = 72.39%, P<0.001, **Figure 5B**).As presented in **Figure 6A**, the meta-analysis conducted under the random - effect model revealed that the pooled SD rate across eleven trials, which included a total of 1128 patients, was 37% (95% confidence interval: 31% - 43%, I² = 76.96%, P < 0.001, **Figure 6A**). In a similar vein, by analyzing data from twelve trials involving 1246 patients with DPM, the meta - analysis using the random - effect model indicated that the combined PD rate was 31% (95%CI: 24% - 38%, I² = 83.27%, P < 0.001, **Figure 6B**), as depicted in **Figure 6B**.

We conducted subgroup analyses based on regions (Europe, other regions), sample sizes (≥60, <60), research scales (single - center, multi - center), research methods (prospective, retrospective), and the number of treatment lines (1Line, 2Line/Later Line, Any) to further analyze the relationship between PD - 1/PD - L1 and CTLA - 4 immune checkpoint inhibitors and the tumor response rate of patients. As shown in **Table 6** subgroup analysis revealed that the ORR of DPM patients receiving PD - 1/PD - L1 inhibitors and CTLA - 4 inhibitors was significantly better in the following situations: in other regions, when the sample size was less than 60, in single - center studies, in prospective studies, and 1Line treatment. From **Table 7**, we can tell that the DCR of DPM patients in subgroups such as other, sample size less than 60, single - center studies, retrospective studies, and 1Line treatment are greater than that of other subgroups.

**Table 6:** Subgroup analysis of pooled of the objective response rate.

Subgroup	No. of studies	Ratio (95% CI)	Heterogeneity	
			I^2^ (%)	Ph
Region				
Europe	7	21%(18%-25%)	0	0.55
Other	6	33%(26%-40%)	64.45	0.02
Sample				
≥60	8	26% (20%-33%)	82.21	<0.001
<60	5	29% (22%-37%)	28.60	0.23
Research scale				
Single-center	4	32% (23%-40%)	17.44	0.30
multicenter	9	26% (20%-32%)	80.13	<0.001
research methods				
prospective	6	28%(21%-36%)	78.37	<0.001
retrospective	7	26% (20-33%)	65.98	0.01
Number of treatment lines				
1Line	4	32%(24%-41%)	64.23	0.04
2Line/latter Line	6	25%(18%-32%)	63.26	0.02
Any	3	25%(20%-30%)	–	–

**Table 7:** Subgroup analysis of pooled of the disease control rate.

DCRsubgroup	No. of studies	Ratio (95% CI)	Heterogeneity	
			I^2^ (%)	Ph
Region				
Europe	7	58% (47%-69%)	85.69	<0.001
Other	5	72% (64%-78%)	63.64	0.03
Sample				
≥60	7	63% (52%-73%)	91.39	<0.001
<60	5	66% (59%-72%)	0	0.56
Research scale				
Single-center	4	67% (59%-74%)	0	0.45
multicenter	8	63% (53%-72%)	90.05	<0.001
Research methods				
prospective	5	63% (51%-75%)	86.7	<0.001
retrospective	7	64% (53%-75%)	86.03	<0.001
Number of treatment lines				
1Line	4	71% (63%-79%)	63.10	0.04
2Line/latter Line	5	61% (43%-77%)	90.35	<0.001
Any	3	59% (53%-64%)	–	–

### 3.4 Safety

**Figure 7:**
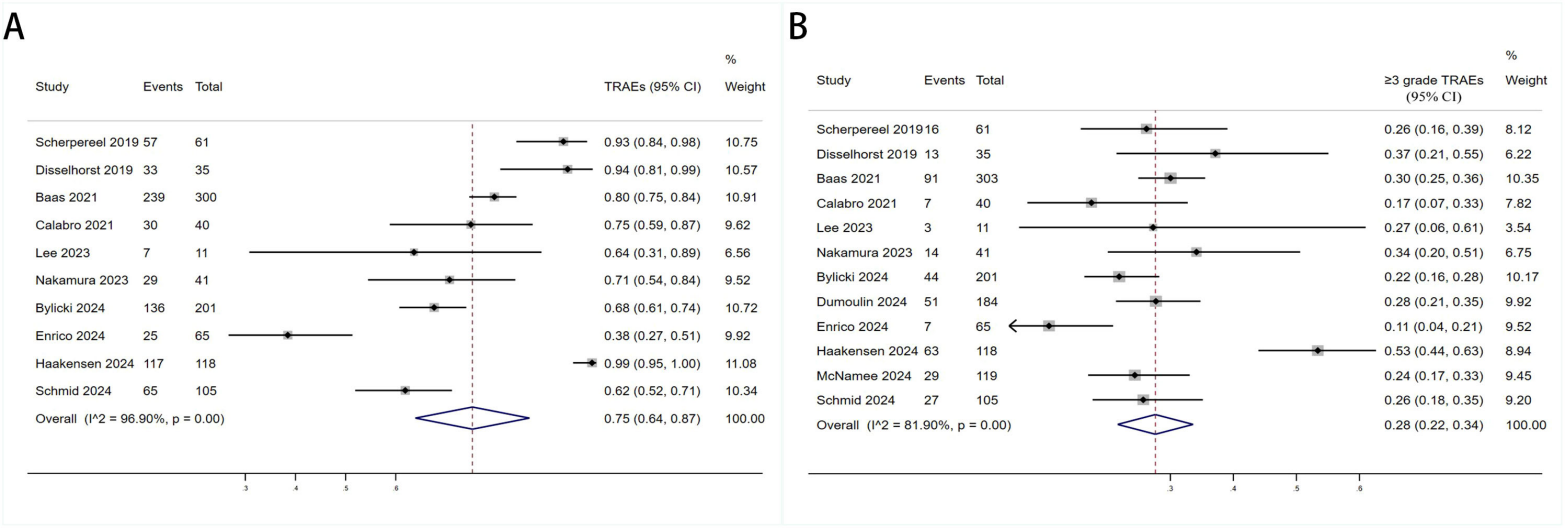
Forest plot for the **(A)** Treatment - Related Adverse Event (TRAE) and **(B)** Progressive Disease (PD) ≥3grade TRAE in DPM patients receiving PD-1/PD-L1 inhibitors combined with CTLA-4 inhibitors.

The TRAEs (any grades and ≥grade 3) associated with anti-PD-1/PD-L1+anti-CTLA-4 in treating DPM were analyzed. Most patients went through grades 1–2 TRAEs and were well tolerated. There were 10 studies with data about any grade TRAEs rate and 12 studies with data about ≥ grade 3 TRAEs. **Figure 7** showed the pooled TRAEs rate was 75% (95%CI:64%-87%, I^2^ = 96.9%, P<0.001, **Figure 7A**) and ≥ grade 3 TRAEs rate was 28% (95%CI:22%-34%, I^2^ = 81.9%, P<0.001, **Figure 7B**).

We conducted a subgroup analysis of the results of TRAEs and ≥ grade 3 TRAEs. According to the subgroup analysis in **Table 8** and **Table 9**, we explored factors such as region, sample size, research scale, research methods and number of treatment lines, which could be an important source of heterogeneity. Europe had a higher incidence of TRAEs (80% vs. 75%) but a less incidence of ≥ grade 3 TRAEs (25% vs. 29%) than other. When sample size <60, TRAEs (79% vs. 77%) and ≥ grade 3 TRAEs (29% vs. 27%) have become more common than sample size≥60. The subgroup analysis also indicated that it was likely that multicenter had a higher incidence of TRAEs (79% vs. 77%) but a less incidence of ≥ grade 3 TRAEs (27% vs. 29%) than single-center. The prospective subgroup showed a clearly higher incidence of TRAEs (88% vs. 60%) and ≥grade 3 TRAEs (32% vs. 23%). According to number of treatment lines,1Line group had lower ratio of AEs (61% vs. 66% vs. 88%) than Any and 2Line/latter Line.

**Table 8:** Subgroup analysis of pooled of the Any TRAEs.

Subgroup	No. of studies	Ratio (95% CI)	Heterogeneity	
			I^2^ (%)	Ph
Region				
Europe	5	80% (65%-91%)	89.63	<0.001
Other	5	75% (49%-94%)	96.45	<0.001
Sample				
≥60	6	77% (59%-91%)	96.63	<0.001
<60	4	79% (63%-91%)	69.92	0.02
Research scale				
single-center	4	79% (63%-91%)	69.92	0.02
multicenter	6	77% (59%-91%)	96.63	<0.001
research methods				
prospective	6	88% (85%-97%)	90.99	<0.001
retrospective	4	60% (47%-72%)	83.86	<0.001
Number of treatment lines				
1Line	3	61% (28%-90%)	–	–
2Line/latter Line	5	88% (69%-99%)	95.32	<0.001
N	2	66% (58%-73%)	–	–

**Table 9:** Subgroup analysis of pooled of the ≥3 grade TRAEs.

Subgroup	No. of studies	Ratio (95% CI)	Heterogeneity	
			I^2^ (%)	Ph
Region				
Europe	6	25% (21%-29%)	9.61	0.35
Other	6	29% (18%-42%)	88.52	<0.001
Sample				
≥60	8	27% (20%-35%)	85.97	<0.001
<60	4	29% (19%-39%)	30.94	0.23
Research scale				
single-center	4	29% (19%-39%)	30.94	0.23
multicenter	8	27% (20%-35%)	85.97	<0.001
Research methods				
prospective	7	32% (23%-41%)	79.99	<0.001
retrospective	5	23% (17%-29%)	59.53	0.04
Number of treatment lines				
1Line	3	21% (8%-39%)	–	–
2Line/latter Line	5	34% (21%-49%)	88.28	<0.001
Any	4	25% (21%-30%)	0	0.61

## 4 Discussion

The advent of T-cell-centered immunotherapies, such as PD-1 and CTLA-4 inhibitors, has significantly reshaped treatment paradigms across various cancers. However, only a select few tumor types exhibit sensitivity to single-agent immune checkpoint inhibition, and the underlying biological mechanisms driving these responses remain poorly understood (47). A review of clinical trials using Tremelimumab monotherapy in diffuse pleural mesothelioma demonstrated an objective response rate (ORR) in the modest range of 3%-13%, with a median progression-free survival (mPFS) of 6.2 months and median overall survival (mOS) of 11 months (48). Similarly, a single-arm study by Josine et al., which treated 34 patients with recurrent malignant pleural tumors using nivolumab, reported a median PFS of 2.6 months (95% CI: 2.23–5.49) and median OS of 11.8 months (95% CI: 9.7–15.7). Other studies on single-agent immunosuppressants in DPM have shown comparable efficacy, though generally lower than the pooled data (49, 50). The low tumor mutational burden (TMB) in DPM (51) and the sparsity of anti-tumor immune cells in its tumor microenvironment (TME) (52) likely contribute to the relatively limited clinical benefit of immune checkpoint inhibitor monotherapy in this patient population.

In our meta-analysis, the combination of PD-1/PD-L1 and CTLA-4 inhibitors yielded a pooled median OS (mOS) of 15.17 months (95% CI: 11.25–19.09) and mPFS of 5.84 months (95% CI: 4.20–7.48). The pooled ORR and disease control rate (DCR) were 27% (95% CI: 22%-32%) and 64% (95% CI: 56%-71%), respectively. Prior to the introduction of dual immune checkpoint inhibitors, the first-line treatment for unresectable DPM involved platinum-pemetrexed chemotherapy. In the landmark CheckMate 743 trial, the chemotherapy group exhibited a mOS of 14.1 months (95% CI: 12.4–16.2) and mPFS of 7.2 months (95% CI: 6.9–8.0), with an ORR of 43% and a DCR of 85%. Similarly, Vogelzang’s phase 3 study reported an mOS of 12.1 months and mPFS of 5.7 months with platinum-pemetrexed, with an ORR of 41.3%. An Italian study evaluating ramucirumab-gemcitabine in 164 mesothelioma patients found a median OS of 13.8 months (53), also lower than the mOS of 15.17 months observed with dual checkpoint blockade. These findings suggest that dual checkpoint inhibition with PD-1/PD-L1 and CTLA-4 inhibitors offers superior overall survival compared to platinum-pemetrexed chemotherapy, though it remains inferior in mPFS, ORR, and DCR. Imaging of DPM is complicated by the diffuse nature of the disease and indistinct tumor margins (54), making overall survival a more reliable and objective endpoint for evaluating efficacy in this context (26). The combination of PD-1/PD-L1 and CTLA-4 inhibitors, however, provides significant long-term survival benefits. Notably, the 1-year OS rate of 60% (95% CI: 53%-67%) and 1-year PFS rate of 29% (95% CI: 24%-34%) support the durability of clinical benefit in certain patients. While these figures are numerically lower than those seen in hypermutated malignancies like melanoma (55), they represent a notable improvement over traditional cytotoxic regimens in DPM, a tumor class historically resistant to systemic therapies. The observed survival benefit is likely driven by the synergistic activation of T-cells via dual blockade of the PD-1/PD-L1 and CTLA-4 pathways, potentially enhancing T-cell priming, infiltration, and tumor microenvironment modulation (56).

As anticipated, the incidence of any-grade treatment-related adverse events (TRAEs) (75%) and grade ≥3 TRAEs (28%) exceeded those reported for monotherapy and chemotherapy (26, 48). These events, primarily immune-related adverse effects, can involve multiple organ systems, including the gastrointestinal, hepatic, pulmonary, and musculoskeletal systems. However, our understanding of their pathogenesis remains incomplete (57). Despite the high incidence of TRAEs, only a small percentage of patients discontinued therapy due to clinically relevant toxicity, with most adverse events being reversible with systemic glucocorticoids and managed safely. Furthermore, when adjusted for exposure, the incidence of TRAEs associated with nivolumab and ipilimumab was lower than with chemotherapy. Interestingly, both the CheckMate-743 trial and a real-world study by Enrico et al. found that patients who discontinued treatment due to TRAEs had a better median OS (26, 43). We hypothesize that the occurrence of TRAEs may correlate with drug dosage, treatment cycle number, and patient health status. These observations emphasize the importance of careful patient selection, proactive monitoring, and multidisciplinary management to mitigate the risk of severe toxicity.

The sequencing of dual immune checkpoint inhibitors (e.g., anti-PD-1/PD-L1 and anti-CTLA-4) with chemotherapy remains an area of active research (58). The enhanced benefit in non-epithelioid DPM may reflect chemotherapy’s limited efficacy in this subtype (10), establishing nivolumab/ipilimumab as preferred first-line for unresectable non-epithelioid disease. For epithelioid DPM, first-line optimal therapy remains undefined. Our subgroup analysis suggests first-line dual immunotherapy outperforms later-line regimens in survival, response, and safety across histologies. However, these findings warrant cautious interpretation given small sample sizes and retrospective design in some studies. Additionally, prospective trials reported better outcomes than real-world studies (43), likely reflecting the latter’s inclusion of more ECOG 2 and non-epithelioid patients. Due to limited histology-stratified reporting, meta-regression was unfeasible; future studies should prioritize histology-specific analyses to guide personalized therapy.

## 5 Limitations

Several limitations must be acknowledged. First, the sample sizes of most included studies were small, limiting statistical power for subgroup analyses. Second, our analysis relied on aggregated data from published articles rather than individual patient-level data, potentially introducing biases in data interpretation. Third, efficacy comparisons across heterogeneous clinical settings (e.g., first-line vs. later-line therapy) introduce complexity; while subgroup analyses suggested superior outcomes with first-line treatment (**Tables 3**–**7**), variability in prior therapies, dosing schedules, and patient comorbidities may confound these results. Fourth, the lack of biomarker-driven patient stratification (e.g., PD-L1 expression, tumor mutational burden) precludes identification of predictive response factors. Therefore, future large-scale prospective trials with standardized treatment protocols and biomarker assessment are needed to validate the efficacy and safety of PD-1/CTLA-4 inhibitor combinations in diffuse pleural mesothelioma.

## 6 Conclusion

In summary, our meta-analysis demonstrates that dual PD-1/PD-L1 and CTLA-4 blockade provides clinically meaningful survival benefits with acceptable safety in DPM, supporting its application in clinical practice. Critically, future research must prioritize biomarker discovery (e.g., PD-L1 expression, tumor mutational burden, inflammatory gene signatures) to identify patients most likely to benefit, enabling personalized therapeutic strategies.

## Data Availability

The datasets presented in this study can be found in online repositories. The names of the repository/repositories and accession number(s) can be found in the article/supplementary material.
